# Endodontic Treatment of a Mandibular Second Premolar with Type IV Wiene's Root Canal: A Case Report

**DOI:** 10.1155/2014/731467

**Published:** 2014-03-03

**Authors:** Noushad Matavan Chalil, Shravan Kini, Sunil Jose, Arun Narayanan, Shahnas Salahudeen, Faizal C. Peedikayil

**Affiliations:** ^1^Department of Conservative Dentistry and Endodontics, Kannur Dental College, Anjarakandy, Kannur, Kerala 670612, India; ^2^Department of Conservative Dentistry and Endodontics, Yenepoya University, Mangalore 575018, India; ^3^Department of Conservative Dentistry and Endodontics, Mahe Institute of Dental Science, Mahe 673310, India; ^4^Department of Periodontics, Kannur Dental College, Anjarakandy, Kannur, Kerala 670612, India; ^5^Department of Pedodontics, Kannur Dental College, Anjarakandy, Kannur, Kerala 670612, India

## Abstract

This case report describes an endodontic treatment of a mandibular second premolar with type IV root canal. A 26-year-old male patient reported pain in right mandibular second premolar. Clinical examination showed a large carious lesion with pulp exposure. Radiographs showed minimal periapical changes and slight widening of periodontal ligament space. Mandibular second premolars usually have one canal. The mandibular second premolar may present large number of anatomic variations. The clinician should be aware of the configuration of the pulp system. This case presents the diagnosis and clinical management of a mandibular second premolar with two distinct canals in the apical third of root (Type IV Wiene's canal configuration), drawing particular attention to tactile examination of all the canal walls and obturating it with calamus 3D obturation system.

## 1. Introduction

Knowledge of basic root and root canal morphology as well as possible variation in anatomy of the root canal system is important in achieving successful nonsurgical root canal treatment (NSRCT). This is followed by negotiation, cleaning and shaping, and obturation of the entire canal system in 3 dimensions [1].

According to Weine a root canal can be present in four types: Type I—single canal from pulp chamber to apex; Type II—two canals leaving the chamber and merging to form a single canal short of the apex; Type III—two separate and distinct canals from chamber to apex; Type IV—one canal leaving the chamber and dividing into two separate apical foramina [2]. The mandibular premolars are difficult to treat as they have a high flare-up and failure rate. It may be due to the extreme variations in root canal morphology. Normally the root canal system of the mandibular second premolar is wider buccolingually than mesiodistally with two pulp horns. At the cervical line the root and canal are oval; this shape tends to become round as the canal approaches the middle of the root. If two canals are present, they tend to be round from the pulp chamber to their foramen. Another anatomic variation is that a single, broad root canal may bifurcate into two separate root canals. Direct access to the buccal canal is usually possible, whereas the lingual canal may be very difficult to find. The lingual canal tends to diverge from the main canal at a sharp angle. In addition, the lingual inclination of the crown tends to direct files buccally, making location of a lingual canal orifice more difficult [3].

This case report describes the successful diagnosis and treatment of mandibular second premolar with a Type IV Weine's configuration.

## 2. Case Report

**Figure 1:**
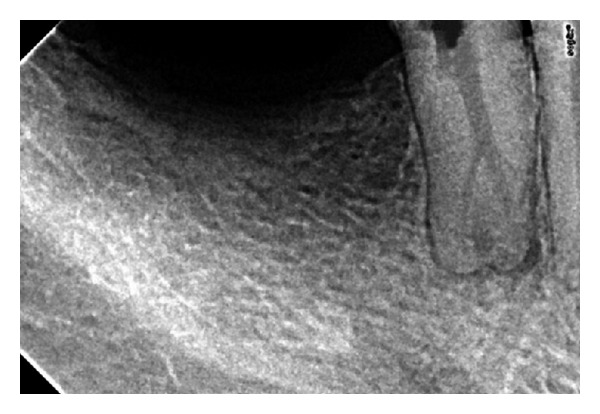
Bifurcated canal and widening of periodontal ligament space.

A 26-year-old male with a noncontributory medical history sought treatment at Mahe Institute of Dental Sciences. The chief complaint was “pain on chewing.” Clinical examination showed a large carious lesion with pulp exposure. The tooth was not sensitive to cold testing or electronic pulp testing (Vitality Scanner, Analytic Technology, Glendora, CA, USA). Investigations for swelling, sinus tract, and periodontal involvement were negative; the pulp was diagnosed as necrotic. Preoperative radiographs revealed minimal periapical changes and slight widening of periodontal ligament space (Figure 1).

**Figure 2:**
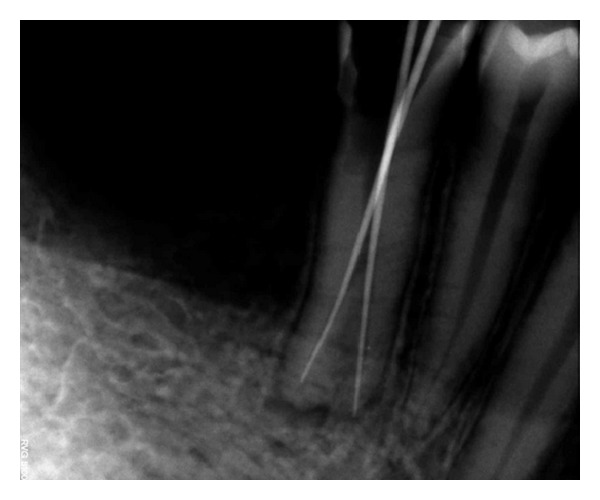
Periapical radiograph showing the working length.

Anaesthesia was obtained and an access cavity was prepared. In the floor of pulp chamber only single orifice was detected. Even with the exploration of the access cavity, no other orifices were found. Using K-file size number 15 (Dentsply, Maillefer, Switzerland) the working length was determined radiographically. This radiograph revealed a vague outline in the apical third which indicated canal splitting (Figure 2). Access was further widened and a number-15 file with severe precurve in the apical third was placed alongside of the first file and radiograph was taken again. This radiograph confirmed Type IV Weine's configuration.

Then biomechanical preparation was carried out with 5.25% NaOCl as the irrigant and canals were prepared with protaper files till No 1 master apical file. The canals were irrigated with saline and 0.2% chlorhexidine. The canals were then dried with sterilized paper points, and calcium hydroxide dressing was given in the canals and the access was closed with Cavit. Patient was recalled after 3 weeks. When patient returned, he was totally comfortable. After removal of Cavit, smear layer was removed and canals were irrigated and dried.

Calamus 3D system was used for obturation. The master cone, protaper F1 gutta-percha, was inserted to the full working length and apical tugback was checked. The master cone is typically cut back about 1.0 mm from the radiographic terminus (RT) so that its most apical end is just short of the “apical constriction.” Three manual pluggers of diameters 0.7 mm, 0.9 mm, and 1.3 mm (Dentsply Tulsa Dental Specialties) were selected to compact the gutta-percha in coronal, middle, and apical thirds, respectively.

The Calamus Pack handpiece was activated to sear off the nonuseful portion of the master cone. During this procedure there is transfer of heat in the coronal 3-4 mm of gutta-percha. A large prefit plugger generates the first WOC (wave of compaction) and automatically compacts warm gutta-percha vertically and laterally into the root canal system.

The pack handpiece was activated again; the EHP (electrically heated plugger) was plunged to 3-4 mm of the previously compacted material, deactivated, and then removed, along with a “bite” of gutta-percha. The medium prefit plugger carries a second wave of condensation and compact middle third of root canal system. This procedure was repeated for apical third of root canal. The downpack was now completed in both main portion and also split portion of the canal. The apical third obturation was now complete.

**Figure 3:**
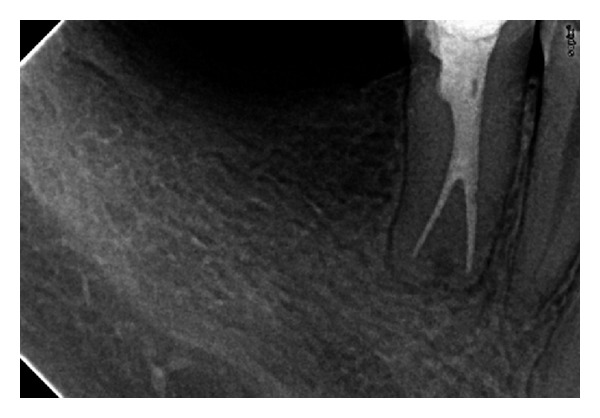
Periapical radiograph showing obturated canals.

The backfill phase was started by dispensing a longer 3 to 4 mm segment of warm gutta-percha into middle region of the canal. The working end of the medium size prefit plugger is stepped circumferentially around the preparation to clean the dentinal walls and flatten the dispensed material. Utilizing the plugger in this manner will promote successful hydraulics and generate “reverse” waves of condensation. The backfill phase was continued till the entire canal was filled. Different horizontally angulated posttreatment radiographs may be taken to confirm the root canal system has been densely obturated, laterally and vertically, to the canal terminus (Figure 3).

## 3. Discussion

Brescia considered that morphology of mandibular premolar was the most variable in the entire dentition. Vertucci (1978) reported that mandibular premolars have Type 1 canal in 70% of the cases, Type 11canal in 4% of the cases, Type 111canal in 1.5 % of the cases and Type 1V canal in 24% of the cases [4].

Hence, it is recommended that clinicians should consider a thorough assessment of radiographs. The crucial step in finding the split canal was tactile examination of main canal with a small, precurved K-file tip [5]. After locating the canals, access was widened to facilitate root canal preparation. Root canal was prepared initially with hand files up to size number 20 and then continued with protaper files.

The calamus 3D obturation system was used for the obturation. The major benefits of this system are that Calamus Dual brings the flow and the pack together in one convenient, space-saving system. The downpack phase creates an effective apical plug and the backfill phase effectively seals lateral canals and furcal canals.

During the pack phase thermo-softened gutta-percha is moved into the narrowing cross-sectional diameters of the preparation and generates a piston effect on the entrapped sealer to fill canals laterally as well as create good apical corkage. During this heating and compaction cycle, the operator will tactilely feel the warm mass of gutta-percha beginning to stiffen as it cools. Importantly, using a plugger to press on warm gutta-percha during the cooling cycle has been shown to completely offset shrinkage [6].

The backfilling of canal is started by activation of flow handpiece. A short 2 to 3 mm segment of warm gutta-percha is dispensed into the most apical region of the empty canal. Small prefit pluggers are used to compact the warm gutta-percha in the middle third. This generates reverse wave of compaction. The backfill procedure is continued till the entire canal is filled [6].

## 4. Conclusion

Clinician should be aware of variations related to canal configuration and types in mandibular first premolars. Tactile examination is a key step in locating the extra/split canal. Three-dimensional obturation not only seals the apical third but also seals multiple portals of exit, that is, the accessory canals and furcal canals.
